# Blockade of interleukin-6 signaling inhibits the classic pathway and promotes an alternative pathway of macrophage activation after spinal cord injury in mice

**DOI:** 10.1186/1742-2094-9-40

**Published:** 2012-02-27

**Authors:** Alexander Rodriguez Guerrero, Kenzo Uchida, Hideaki Nakajima, Shuji Watanabe, Masaya Nakamura, William EB Johnson, Hisatoshi Baba

**Affiliations:** 1 Department of Orthopaedics and Rehabilitation Medicine, Faculty of Medical Sciences, University of Fukui, Matsuoka-Shimoaizuki 23, Eiheiji, Fukui 910-1193, Japan; 2 Servicio de Neurocirugia, Hospital Nacional Rosales, Universidad de El Salvador, San Salvador, El Salvador; 3 Department of Orthopaedic Surgery, School of Medicine, Keio University, Tokyo 160-8582, Japan; 4 Life & Health Sciences, Aston University, Aston Triangle, Birmingham B4 7ET, UK

**Keywords:** Spinal cord injury, Interleukin (IL)-6/IL6 receptor (R), Alternatively activated macrophage, Arginase 1, Inducible nitric oxide synthase (iNOS), T helper (Th) cytokine

## Abstract

**Background:**

Recent *in vivo *and *in vitro *studies in non-neuronal and neuronal tissues have shown that different pathways of macrophage activation result in cells with different properties. Interleukin (IL)-6 triggers the classically activated inflammatory macrophages (M1 phenotype), whereas the alternatively activated macrophages (M2 phenotype) are anti-inflammatory. The objective of this study was to clarify the effects of a temporal blockade of IL-6/IL-6 receptor (IL-6R) engagement, using an anti-mouse IL-6R monoclonal antibody (MR16-1), on macrophage activation and the inflammatory response in the acute phase after spinal cord injury (SCI) in mice.

**Methods:**

MR16-1 antibodies versus isotype control antibodies or saline alone were administered immediately after thoracic SCI in mice. SC tissue repair was compared between the two groups by Luxol fast blue (LFB) staining for myelination and immunoreactivity for the neuronal markers growth-associated protein (GAP)-43 and neurofilament heavy 200 kDa (NF-H) and for locomotor function. The expression of T helper (Th)1 cytokines (interferon (IFN)-γ and tumor necrosis factor-α) and Th2 cytokines (IL-4, IL-13) was determined by immunoblot analysis. The presence of M1 (inducible nitric oxide synthase (iNOS)-positive, CD16/32-positive) and M2 (arginase 1-positive, CD206-positive) macrophages was determined by immunohistology. Using flow cytometry, we also quantified IFN-γ and IL-4 levels in neutrophils, microglia, and macrophages, and Mac-2 (macrophage antigen-2) and Mac-3 in M2 macrophages and microglia.

**Results:**

LFB-positive spared myelin was increased in the MR16-1-treated group compared with the controls, and this increase correlated with enhanced positivity for GAP-43 or NF-H, and improved locomotor Basso Mouse Scale scores. Immunoblot analysis of the MR16-1-treated samples identified downregulation of Th1 and upregulation of Th2 cytokines. Whereas iNOS-positive, CD16/32-positive M1 macrophages were the predominant phenotype in the injured SC of non-treated control mice, MR16-1 treatment promoted arginase 1-positive, CD206-positive M2 macrophages, with preferential localization of these cells at the injury site. MR16-1 treatment suppressed the number of IFN-γ-positive neutrophils, and increased the number of microglia present and their positivity for IL-4. Among the arginase 1-positive M2 macrophages, MR16-1 treatment increased positivity for Mac-2 and Mac-3, suggestive of increased phagocytic behavior.

**Conclusion:**

The results suggest that temporal blockade of IL-6 signaling after SCI abrogates damaging inflammatory activity and promotes functional recovery by promoting the formation of alternatively activated M2 macrophages.

## Background

Spinal cord injury (SCI) is followed by disruption of the blood-brain barrier and influx of inflammatory cells, a process facilitated by proteolytic and oxidative enzymes, and various pro-inflammatory cytokines. The pro-inflammatory cytokines are produced by resident microglia, along with infiltrating neutrophils and macrophages, and induce a reactive process of secondary cell death in the tissue surrounding the original site of injury [1-3]. This secondary damage continues in the days and weeks following SCI, which may lead to increase in cavitation and glial scar formation at the lesion site, exacerbating neurological dysfunction [4-6]. Evidence suggests that such inflammation may be beneficial; for example, macrophages phagocytose the myelin debris present in the injured spinal cord, which is known to inhibit axonal regeneration [7-9], and they also release protective cytokines such as basic fibroblast growth factor, nerve growth factor and neurotropin-3, which promote neuronal regeneration, and tissue repair [10]. Indeed, implantation of activated macrophages after SCI is reported to promote axonal regeneration [11]. However, macrophages can also have adverse effects on damaged neural tissues, including excessive inflammation, axonal retraction, and axonal die-back [12-14], and the depletion of hematogenous macrophages after SCI can promote functional recovery [15]. Such variation in the effects of macrophages could be the result of the presence of different activation pathways for the locally present macrophages, possibly generating sub-populations of cells with divergent abilities [16,17].

Recent studies have indicated that different macrophage sub-populations can arise during the immunological and inflammatory responses to various conditions, based on their phenotypes [18,19]. This divergence is referred to as macrophage polarization, and it has been reported both in non-neural [20] and in neural tissue [21,22], and in *in vitro *and *in vivo *experiments [23]. Two subtypes of macrophages have attracted great interest in the field of SC regeneration: classically activated (M1 phenotype) and alternatively activated (M2 phenotype) macrophages [24-27]. Whereas classically activated macrophages are the product of exposure to T helper (Th)1 cytokines, such as interferon (IFN)-γ and tumor necrosis factor (TNF)-α, alternatively activated macrophages are activated via Th2 cytokines, such as interleukin (IL)-4, IL-10, and IL-13, which lead to an M2 phenotype that has enhanced phagocytic behavior and anti-inflammatory roles [21,24,28,29]. Alternatively activated macrophages are involved in the recovery of SCI [22,30]. Thus, modification of the SCI microenvironment to increase the number of these alternatively activated macrophages may have a neuroprotective effect.

The inflammatory process itself is necessary for SC regeneration [31,32], but it is characterized by increased expression of various pro-inflammatory cytokines that promote the activation of macrophages through the classic pathway [3,6]. One of these cytokines, IL-6, is an early and key factor that triggers the inflammatory response after SCI [33-35]. IL-6 itself can enhance the expression of other inflammatory cytokines, including TNF-α and IL-1β [33,36], which regulates the entrance of neutrophils into the injured SC [18,37]. These neutrophils are the first cells to enter the lesion after SCI [32,38], and they form an important source of IFN-γ [39], along with other infiltrating macrophages and resident microglia [40,41]. Complete blockade of IL-6 has detrimental effects on recovery from SCI, perhaps reflecting the requirement of inflammation for SC regeneration [42]. However, a temporal blockade of IL-6 has been shown to downregulate the expression of other pro-inflammatory cytokines in the acute phase of SCI, while preserving the beneficial effects of inflammation in the later stages of recovery [43,44]. Interestingly, IL-6 blockade increases the number of microglia recruited to the injured SC of [45]. Microglia are the major source of the Th2 cytokines IL-4 [46] and IL-13 [47] in the normal central nervous system, although neutrophils and macrophages also act as a source of IL-4 [48,49].

Based on these previous findings, we hypothesized that temporal blockade of IL-6 might modify the acute phase inflammatory response after SCI to create an alternative activating environment at the site of injury, which may promote the generation of M2 phenotype macrophages. The present study was thus designed to investigate the effects of temporal blockade of IL-6/IL-6 receptor (IL-6R) engagement (see Additional file 1) during this response, using a rat anti-mouse IL-6R monoclonal antibody (MR16-1) [50], focusing particularly on potential effects of IL-6 blockade on the different pathways of macrophage activation.

## Methods

### Animal model of spinal cord injury

Experiments were conducted in a total of 211 adult male Jcl:ICR mice (Clea, Tokyo), aged 10 weeks, with a mean body weight of 41.2 ± 3.2 g (± SD). They were assigned to the following groups: MR16-1 treated group (n = 85), rat IgG control group (n = 85), saline control group (n = 25), sham group (n = 21) and naive group (n = 5). Mice were anesthetized with isoflurane (Forane^®^; Abbot Japan, Tokyo, Japan), and complete laminectomy was performed at the level of the 10th thoracic vertebra under a surgical microscope (VANOX-S; Olympus, Tokyo, Japan), after exposing the dorsal surface of the dura mater and taking the utmost care in avoiding any dural tear. A contusion SCI was produced using a commercially available SCI device (Infinite Horizons Impactor; Precision Systems and Instrumentation LLC, Fairfax, VA, USA) with an impact force of 60 kilodynes [51]. Immediately after injury, the subjects in the treatment group received a single intraperitoneal dose (50 μg/g body weight) of MR16-1 antibody (Chugai, Tokyo, Japan), and the control groups received either a single dose of rat IgG control antibodies in the same volume and concentration (lot number 918719; Abcam plc, Cambridge, Cambridgeshire, UK) [43,44,52] or a single dose of saline in the same volume [53]. Mice that underwent only laminectomy served as the sham-operated group.

After surgery, the mice were maintained in an isothermic cage until recovery. They were then transferred to a bacteria-free biologically clean room set on a12-hour light/dark cycle and provided with food and water *ad libitum*. Each mouse received manual bladder expression twice daily until the recovery of sphincter control. The experimental protocol was approved by the Ethics Committee for Animal Experimentation of Fukui University.

### Immunohistochemistry

Once deep anesthesia was achieved, transcardial perfusion was performed (sham n = 16 mice, rat IgG control group n = 45 mice, MR16-1 treated group n = 45 mice), followed by fixation with 4% paraformaldehyde in 0.1 mol/l phosphate-buffered saline (PBS). The spinal cords were dissected out and post-fixed in the same fixative for a few hours. The tissue samples were immersed in 10% sucrose in 0.1 mol/l PBS at 4°C for 24 hours, and 30% sucrose in 0.1 mol/l PBS for 24 hours. Segments of the SC (cord segments T8 to T12) were embedded in optimal cutting temperature compound (Sakura Finetek, Torrance, CA, USA) and cut on a cryostat into serial axial or sagittal frozen sections 10 μm thick. The sections were serially mounted on glass slides, and fixed with 2% paraformaldehyde in 0.1 mol/l PBS for 5 minutes, rinsed in PBS, and stored at -80°C. Luxol fast blue (LFB) staining was used to evaluate the spared myelin and extent of demyelination.

For immunofluorescence staining with fluorescent antibodies, frozen sections were permeabilized with 0.1 mol/l Tris-HCl buffer (pH 7.6) containing 0.3% Triton X-100. The following primary antibodies were diluted in commercial diluent (Antibody Diluent with Background Reducing Components; Dako Cytomation, Carpenteria, CA, US) and applied overnight at 4°C: rabbit anti-integrin αM (equivalent to CD11b), 1:200 dilution; goat anti-arginase 1,1:200; rat anti-CD16/32, 1:200 and goat anti-CD206, 1:200 (all Santa Cruz Biotechnology, Santa Cruz, CA, USA); rabbit anti-inducible nitric oxide synthase (iNOS), 1:200 (BD Pharmingen, San Jose, CA, USA); and mouse anti-neurofilament 200 kDa (NF-H), 1:500; and mouse anti-growth-associated protein (GAP-43), 1:500 (both Abcam plc). The sections were then incubated for 1 hour at room temperature with Alexa fluor-conjugated 488 or 568 secondary antibody, 1:250 (Molecular Probes, Eugene, OR, USA). Some sections were also counterstained with the nuclear marker DAPI (Abbott Molecular, Des Plaines, IL, USA). The sections were then washed, wet-mounted, and examined by epifluorescence. All images were obtained using a fluorescence microscope (Olympus AX80; Olympus Optical, Tokyo, Japan) or a confocal laser scanning microscope (model TCS SP2; Leica Instruments, Nussloch, Germany), where the 405, 488 and 543 nm lines of the argon/helium-neon laser were used for fluorescence excitation.

### Semi-quantitative analysis of tissue staining

For semi-quantitative analysis of demyelination, 10 axial sections randomly selected at a distance up to 5 mm cephalad and caudal to the epicenter were stained with LFB and examined at 14 and 42 days after SCI. The LFB-positive area in the ventrolateral funiculus was analyzed under ×400 magnification using grain counting with the light intensity automatically set by the color image analyzer (MacSCOPE; Mitani, Fukui, Japan). The LFB-positive area in which the density significantly exceeded the threshold of each background was calculated as the percentage cross-sectional area of residual tissue, as described previously [54].

To quantify the NF-H-positive and GAP-43-positive areas at the aforementioned time points, the following procedure was performed; 3 mid-sagittal sections through the injured portion of the SC were selected randomly, and 20 high-magnification (×400) non-overlapping photomicrographs of each injury epicenter were selected using a confocal microscope (TCS SP2; Leica). Using color image analyzer software (MacSCOPE; http://www.mitani-visual.jp/en/macscope01.html), a threshold was automatically calculated from the basal fluorescence of samples of intact tissue. The light intensity of the injured samples was calculated as grain counting. The area with light intensity exceeding the threshold set by the program was automatically counted as positive, and was expressed as units of positive area [55].

To semi-quantify the number of CD11b-positive cells double-immunostained with iNOS, arginase 1, CD16/32 and CD206 at 3 hours and 1, 3, 7, and 14 days post-injury, the following procedure was performed: 5 axial sections at the injury epicenter were selected at random, with 20 non-overlapping high-magnification photomicrographs (×400 magnification, under confocal microscope; TCS SP2; Leica) were taken per section. The numbers of merged and non-merged CD11b-positive cells were automatically counted by MacSCOPE, and the average number of positive cells in all the microphotographs was calculated. The basal fluorescence was obtained using the non-injured portions of the sham-SC samples that were incubated without primary antibodies; the procedure was performed for each antibody (n = 20 axial sections from each injury epicenter) using adjacent sections. The light intensity and threshold values were maintained at constant levels when collecting the digitized images in all analysis.

### Assessment of locomotor behavior

Hind-limb motor function was evaluated using the Basso Mouse Scale (BMS) open field locomotor test, in which the scores range from 0 points (no ankle movement) to 9 points (complete functional recovery) [56]. BMS scores were recorded at 1, 3, 7, 14, 21, 28, 35, and 42 days after SCI, by two independent examiners blinded to the experimental conditions (HN, SW). We assessed hind-limb motion, mainly to evaluate coordinated movement and stepping. When differences in the BMS score between the right and left hind limbs were detected, we took the average of the two scores.

### Immunoblot analysis

Immediately after deep anesthesia, the damaged SC around the epicenter of the lesion (5 mm in length) was carefully dissected *en bloc *from the thoracic spine, and stored immediately at -80°C in liquid nitrogen. Segments were separated by centrifugation at 15,000 *g *for 30 s using a commercial kit (BioMasher Rapid Homogenization Kit; Funakoshi, Tokyo, Japan), then solubilized in 1× RIPA lysis buffer (Santa Cruz Biotechnology), homogenized, and stored at -80°C. The protein concentration in the obtained samples was determined by a Lowry protein assay using a DC protein assay kit (Bio-Rad Laboratories, Hercules, CA, USA). Samples containing the protein mixtures in Laemmli sodium dodecylsulfate buffer were boiled and subjected to immunoblot analysis. Total protein (20 μg/lane) was separated on 12.5% SDS-PAGE and transferred onto polyvinylidene difluoride membrane (PE Applied Biosystems, Foster City, CA, USA) for 70 min using a semi-dry blot apparatus.

The membrane was washed twice in PBS containing 0.05% Tween 20, blocked by 5% skimmed milk in PBS for 1 hour at room temperature, and then incubated overnight at 4°C with one of the following antibodies: rabbit anti-TNF-α, 0.2 μg/ml; rabbit anti-IFN-γ,0.2 μg/ml (both Abcam plc); rat anti-IL-4, 1:200; or goat anti-IL-13, dilution 1:200 (both Santa Cruz Biotechnology). After three washes in 0.1 mol/l PBS, the membranes were incubated for 1 hour in the respective secondary IgG/horseradish peroxidase complex antibodies: anti-goat 1:1000, anti-rabbit 1:5000, or anti-rat 1:1000 (all Santa Cruz Biotechnology). After three washes in 0.1 mol/l PBS, a commercial detection kit (ECL Advance Western Blot Detection kit; GE Healthcare, Amersham, Buckinghamshire, UK) was used for 1 minute, and the membrane then exposed to X-ray film for visualization of peroxidase activity and thus determination of the level of each specific protein. The band intensities were normalized to β-actin 1:2000 (Abcam plc), and commercial standards (Kaleidoscope Prestained Standards; Bio-Rad Laboratories) were used as molecular weight controls. To exclude increased inflammatory response secondary to the use of the rat IgG control antibodies, a second control group using saline alone was also included in this experiment (saline control group n = 15, rat IgG control group n = 15, MR16-1 treated group n = 15).

### Flow-cytometry analysis

Immediately after deep anesthesia, the mouse (naive group n = 5, sham group n = 5, rat IgG control group n = 25, MR16-1 treated group n = 25) was intracardially perfused with 200 ml of ice-cold 0.1 mol/l PBS, and the SC was harvested. The damaged SC around the epicenter of the lesion (6 mm in length) was surgically dissected out with 175 U/ml collagenase (Sigma-Aldrich, St. Louis, MO, USA) for 1 hour at 37°C. Cells were washed in DMEM (Invitrogen Life Technologies, Carlsbad, CA, USA) containing 10% fetal bovine serum and filtered through a 40 μM nylon cell strainer (BD Biosciences, San Jose, CA, USA) under centrifugation to remove tissue debris and obtain a single-cell suspension, as described previously [38].

From this point on, a cell-count was performed before every staining in every sample to maintain a cell density of 1.0 × 10^6 ^cells/100 μL. Cells were incubated for 1 hour on ice with the following fluorescent antibodies: allophycocyanin (APC) rat anti-CD45, 0.25 μg/1 ml; Pacific Blue rat anti-Ly-6 G/Ly-6 C, 1.0 μg/1 ml (equivalent to Gr-1 (both BioLegend, San Diego, CA, USA) and PerCP-Cy 5.5 rat anti-CD11b, 0.25 μg/1 ml (BD Pharmigen). For intracellular staining [32], the cells were resuspended in commercial fixation buffer and treated with permeabilization buffer (both Santa Cruz Biotechnology) followed by resuspension in ice-cold PBS and incubation for 1 hour with goat anti-arginase 1, 1:200 conjugated to fluorescein isothiocyanate (FITC), 1:200 (Santa Cruz Biotechnology), phycoerythrin (PE) conjugated rabbit anti-iNOS, 3.0 μg/ml (Abcam plc); PE/Cy7 conjugated rat anti-CD16/32, 1.0 μg/ml or FITC rat anti-CD206, 1.0 μg/ml (both BioLegend). To verify the phagocytic characteristics of the identified macrophages and microglia, we used biotin rat anti-macrophage antigen-2 (Mac-2), 1.0 μg/ml and biotin rat anti-macrophage antigen-3 (Mac-3), 0.25 μg/mlwhich were subsequently labeled with conjugation to streptavidin-APC/Cy7, 0.06 μg/ml (all BioLegend).

A parallel set of samples was incubated with the following intracellular markers: FITC rat anti-IFN-γ, 3 μg/ml (Abcam plc) and goat anti-IL-4, 0.25 μg/ml (Santa Cruz Biotechnology) secondarily conjugated to PE, dilution 1:200 (Santa Cruz Biotechnology). Samples with cells alone were used as negative controls to eliminate background autofluorescence, and samples containing cells incubated with a single added antibody were used as positive control. These were used to set up the cytometer alignment and to remove any spectral overlap. Mixed samples of cells from the spinal cords of the naive and rat IgG control groups were used to identify the region of interest in the light-scatter plot (see Additional file 2).

Flow cytometry was performed using a fluorescence-activated cell sorting (FACS) device (FACS CantoTM II; Becton Dickinson Biosciences, San Jose, CA, USA) using forward scatter to further eliminate any cellular debris from analysis. In each test, a minimum of 250,000 cells were analyzed and the data processed (BD FACSDiva software; Becton Dickinson Biosciences). The different cell populations present in the suspension were classified according to the expression of antigens, as stated in the cited studies: CD45^high^/CD11b^high^/GR-1^high ^neutrophils [57]; CD45^low^/CD11b^high^/GR-1^negative ^microglia [32]; and CD45^high^/CD11b^high^/GR-1^negative ^macrophages [38]. At the aforementioned time points, the populations of neutrophils, microglia, and macrophages were identified, together with their intracellular positivity for IFN-γ and IL-4. The phenotype of macrophage sub-populations was confirmed by the expression of iNOS and CD16/32 (pro-inflammatory M1 macrophages) or arginase 1 and CD206 (anti-inflammatory M2 macrophages). The levels of Mac-2 and Mac-3 immunopositivity in microglia and arginase 1-positive macrophages were quantified (see Additional file 3).

### Statistical analysis

All statistical analyses were carried out by two independent biostatisticians blinded to the groups (HN, SW). All values are expressed as mean ± SD. Differences between groups were examined for statistical significance using either the paired-*t *test or one-way factorial analysis of variance (ANOVA). *P *≤ 0.05 was considered significant (Tukey's *post hoc *analysis). The above tests were conducted using SPSS software (version 13.0; SPSS, Chicago, IL, USA).

## Results

### Anti-interleukin (IL)6-receptor (MR16-1) treatment increased the area of spared myelin, neurofilament heavy 200 kDa-positive and growth-associated protein-43-positive nerve fibers, and locomotor function after spinal cord injury

**Figure 1:**
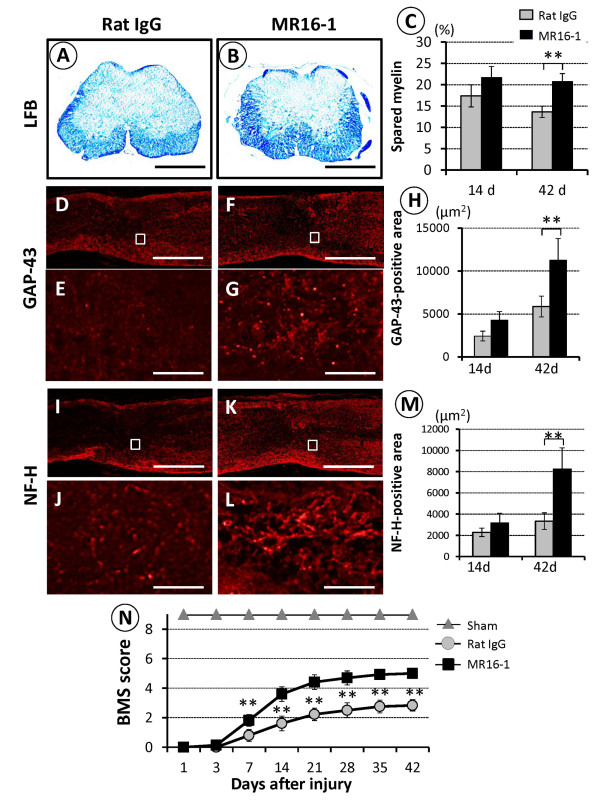
**Histological evaluation and locomotor function after anti-interleukin (IL)-6-receptor (MR16-1) treatment**. (**A**) Compared with the rat IgG control group, axial sections at the epicenter of the injury site obtained at 42 days post-injury and stained with luxol fast blue (LFB) showed a remarkable reduction in the area of demyelination in (**B**) the MR16-1-treated group. (**C**) Quantification of LFB-positive spared myelin areas in the ventrolateral funiculus at the lesion site showed a significant difference between the two groups at 42 days, but not at 14 days post-injury. Representative images of injury epicenter mid-sagittal sections at 42 days after spinal cord injury in (**D**, **I**) the rat IgG control group and (**F**, **K**) MR16-1-treated group: in the high-magnification photomicrographs of the respective boxed area, a greater abundance of neurofilament heavy 200 kDa-positive and growth-associated protein nerve fibers were seen in (**G**, **L**) the MR16-1-treated group compared (**E**, **J**) with the rat IgG control group. (**H**, **M**) Note the significant differences in the GAP-43-positive and NF-H positive areas at 42 days after injury between the two groups. (**N**) Analysis of the locomotor Basso Mouse Scale (BMS) score after SCI. A significant improvement in hind-limb motor function was seen in the MR16-1-treated group compared with the rat IgG control group from 7 days after injury. Scale bar (**A**, **B**) 200 μm, (**D**, **F**, **I**, **K**) 500 μm, (**E**, **G**, **J**, **L**) 50 μm. (**D**-**G**) GAP-43 conjugated to Alexa fluor 568 (red); (**I**-**J**) NF-H conjugated to Alexa fluor 568 (red). Data are expressed as mean ± SD. (**C**, **H**, **M**) n = 3 for each group; (**N**) n = 5 for each group. (**C**, **H**, **M**) paired *t*-test; (**N**) ANOVA test. *P <0.05, **P <0.01

To examine the therapeutic effects of MR16-1 on SC repair, we used LFB staining to evaluate the sparing of myelin sheaths around axons, and the immunoreactivity of both NF-H-positive and GAP-43-positive nerve fibers at the lesion epicenter, at 14 and 42 days after SCI. The area of spared myelin in the MR16-1 treated group was significantly greater than that in the rat IgG control group at 42 days (Figure 1A-C). A significantly larger GAP-43-positive area was also seen in the MR16-1 treated group at 42 days (Figure 1D-H). Similarly, there was a significant difference in the NF-H-positive area between the two groups at 42 days (Figure 1I-M).

The contusive SCI resulted in immediate complete paralysis. At 7 days post-injury, the MR16-1 treated group showed a significant improvement in BMS locomotor score compared with the rat IgG control group, and this difference continued up to 6 weeks post-injury, at which time recovery in both groups reached a plateau (Figure 1N). At that point, the BMS score of the MR16-1 treated group was 5.0 ± 0.3, reflecting consistent plantar stepping with some coordination, whereas that of the control group was 2.8 ± 0.3 points, with extensive ankle movement and dorsal stepping only at the last time point.

### MR16-1 treatment reduced protein levels of interferon (IFN)-γ and TNF-α and increased levels of interleukin (IL)-4 and IL-13 at the lesion site in the acute phase after SCI

**Figure 2:**
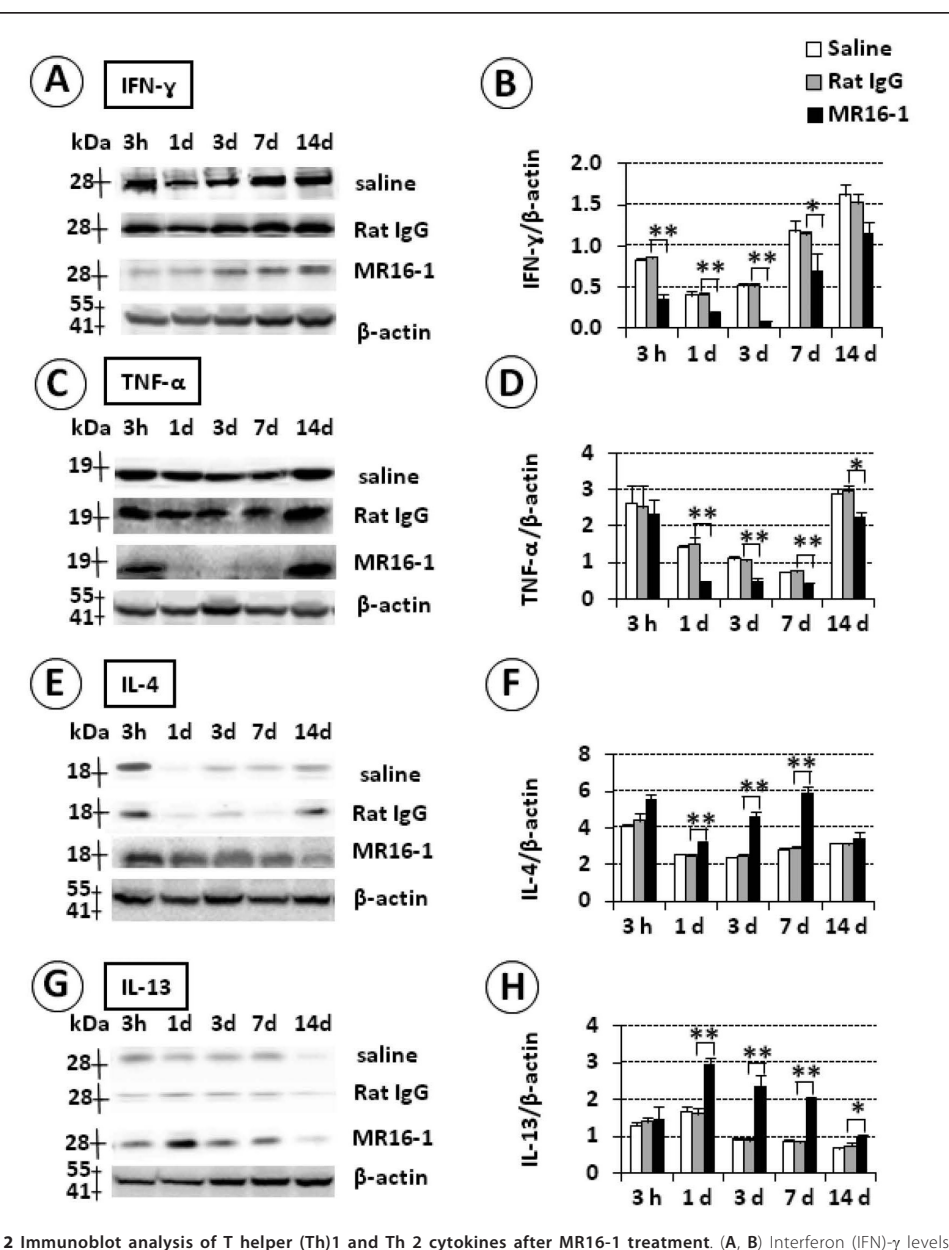
**Immunoblot analysis of T helper (Th)1 and Th 2 cytokines after MR16-1 treatment**. (**A**, **B**) Interferon (IFN)-γ levels were consistently higher in the control groups, and the difference with the MR16-1-treated group was significant up to 7 days post-injury. (**C**, **D**) Tumor necrosis factor (TNF)-α levels were enhanced in the control groups, and were significantly different compared with the MR16-1-treated group from 1 to 14 days post-injury. (**E**, **F**) Interleukin (IL)-4 levels remained significantly higher in the MR16-1-treated group from 1 to 7 days post-injury compared with the controls. (**G**, **H**) IL-13 levels were increased in the MR16-1-treated group, and were significantly different to the control groups between 1 and 4 days post-injury. No difference was found in the cytokine expression between the control groups (saline and Rat IgG). Each graph indicates relative band intensity normalized to that of β-actin. (**B**, **D**, **F**, **H**) Data are expressed as mean ± SD, n = 3 for each group. *P ≤ 0.05, **P ≤ 0.01 by ANOVA

To determine whether MR16-1 treatment affected the inflammatory response in the acute phase after SCI, we evaluated protein levels of IFN-γ and TNF-α (Th1 cytokines) and IL-4 and IL-13 (Th2 cytokines) by immunoblot analysis (Figure 2). IFN-γ levels (28 kDa band) increased in the control groups (rat IgG and saline alone) from 3 hours up to 14 days post-injury, whereas this increase was abrogated in the MR16-1-treated group, with statistical significance up to 7 days post-injury (Figure 2A, B). TNF-α levels (19 kDa band) were stable during the post-injury period in the control groups, but were markedly reduced in the MR16-1-treated group from 1 to 14 days after injury (Figures 2C, D).

By contrast, the protein levels of IL-4 (18 kDa band) initially seen at 3 hours post-injury disappeared rapidly in the control groups by 1 day, and remained relatively low thereafter, whereas they were also increased at 3 hours post-injury in the MR16-1-treated group and remained high, reaching a peak at 7 days post-injury. The differences in IL-4 levels between the control groups and the MR16-1-treated group at 1, 3 and 7 days post-injury were significant (Figure 2E, F). IL-13 levels (13 kDa band) decreased from 1 to 14 days post-injury in the control groups, but were persistently high in the MR16-1-treated group during the same time period, and significantly greater than the control groups from 1 day post-injury and thereafter (Figure 2G, H). No further significant differences in the protein levels of these cytokines were detected between the MR16-1-treated group and the control groups at 14 days after injury. No differences in the protein levels of the studied cytokines were noted between the two control groups, indicating a lack of potentially harmful effects of rat IgG compared with saline.

### MR16-1 treatment was associated with a reduction in M1 macrophages and increased M2 macrophages at lesion site in the acute phase after spinal cord injury

**Figure 3:**
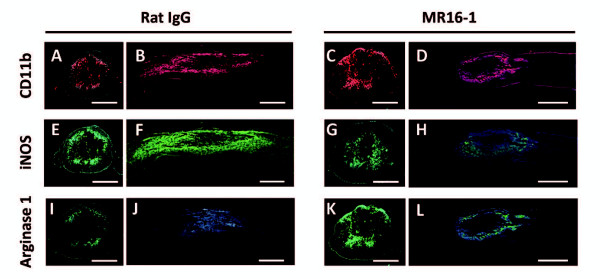
**Distribution of classically activated (M1) and alternatively activated (M2) phenotype macrophages in the injured spinal cord at 3 days after MR16-1 treatment**. A common finding in the MR16-1-treated group was (**C, D**) a focal injury site with centralized presence of CD11b-positive cells, whereas (**A**, **B**) in the rat IgG control group, there was a larger injury site with cephalic and distal expansion of CD11b-positive cells. (**E**, **F**) Inducible nitric oxide synthase (iNOS)-positive cells were distributed over the same CD11b-positive area in the rat IgG control group, whereas a weaker reaction to iNOS was seen in (**G**, **H**) the MR16-1-treated group. (**K**, **L**) Arginase 1-positive cells were localized more specifically to the injury site in the MR16-1-treated group, whereas arginase 1-positive cells were rare in (**I**, **J**) the rat IgG control group. (**A**-**D**) CD11b conjugated to Alexa fluor 568 (red); **E**-**H**) iNOS conjugated to Alexa fluor 488 (green);(**I**-**L**) arginase 1 conjugated to Alexa fluor 488 (green); (**A**-**L**) DAPI used for nuclear counterstaining (blue). Scale bar: (**A**, **C**, **E**, **G**, **I**, **K**) 200 μm,(**B**, **D**, **F**, **H**, **J**, **L**) 500 μm

**Figure 4:**
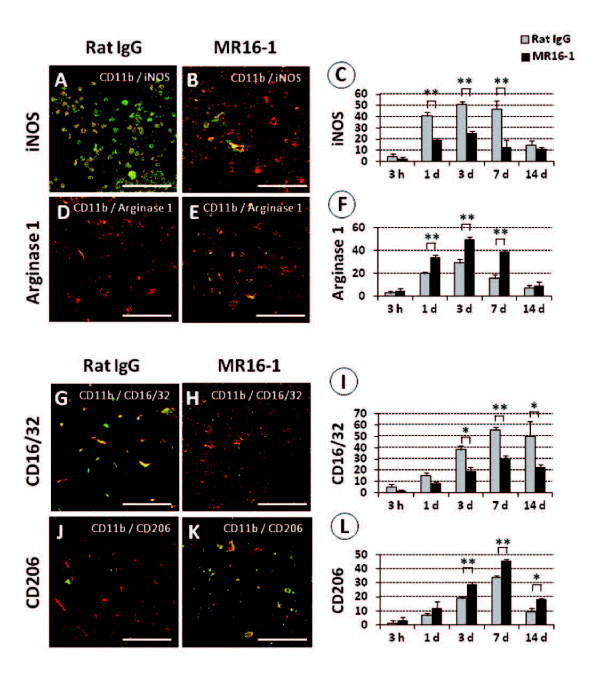
**Effects of MR16-1 treatment on macrophage polarization after spinal cord injury (SCI), as delineated by double immunolocalization**. (**A**) At 3 days post-injury, large amounts of inducible nitric oxide synthase (iNOS) colocalized with CD11b (merged)-positive cells were found inthe rat IgG control group), whereas only a few were found in (**B**) the MR16-1-treated group. (**C**) The differences in the presence of merged double-immunopositive cells between the two groups were significant from 1 to 7 days after injury. (**D**) Scarce numbers of arginase 1 colocalized with CD11b-positive (merged) cells were found in the rat IgG control group, in contrast to (**E**) the MR16-1-treated group; (**F**) analysis showed predominance of merged double-immunopositive cells in the MR16-1-treated group from 1 to 7 days after injury. (**G**) At 7 days post-SCI, a larger number of cells immunopositive for CD16/32 and CD11b (merged) was found in the injury epicenter in the rat IgG control group than in (**H**) the MR16-1-treated group, and (**I) **these differences were significant from 3 to 14 days after injury. (**K**) Cells immunopositive for CD206 and CD11b (merged) were prevalent in the MR16-1-treated group, but barely present in (**J**) the rat IgG control group. (**L) **The population of CD206/CD11b-immunopositive cells became significantly greater in the MR16-1-treated group compared with the control group from 3 to 14 days after injury. CD11b conjugated to (**A**, **B**, **D**, **E**, **G**, **H**, **J**, **K**); Alexa fluor 568 (red) (**A**, **B**) iNOS, (**D**, **E**) arginase 1, (**G**, **H**) CD16/32 and (**J**, **K**) CD206 conjugated to Alexa fluor 488 (green). Scale bar = 50 μm. (**C**, **F**, **I**, **L**) Data are expressed as mean ± SD; n = 3 for each group. *P <0.05, **P <0.01 by paired *t*-test.

To determine whether MR16-1 treatment affects macrophage polarization, we quantified the populations of M1 and M2 phenotypes (Figure 3, Figure 4), and CD11b-positive cells (both types of macrophages). CD11b immunostaining in mid-sagittal sections at 3 days post-injury identified relatively high numbers of positive cells extending proximally and distally from the lesion site (in post-traumatic hematoma) in the rat IgG control group (Figure 3A, B), compared with a more localized distributions of positive cells immediately around the lesion site in the MR16-1-treated group (Figure 3C, D). There was also a greater number and similar distribution of iNOS-positive cells at 3 days post-injury in the rat IgG control group (Figure 3E, F), compared with the MR16-1-treated group (Figure 3G, H). By contrast, arginase 1-positive cells at 3 days post-injury populated the lesion site in the MR16-1-treated group (Figure 3K, L) in higher numbers than in the control group (Figure 3I, J). The patterns of immunopositivity for iNOS and arginase 1 in the rat IgG control group and MR16-1-treated groups, respectively, matched the distribution of CD11b-positive cells.

At 3 days post-injury, representative axial sections of the injured spinal cords at the lesion epicenter were double-immunostained for CD11b plus either iNOS or CD 16/32, or for CD11b plus either arginase 1 or CD206. The MR16-1-treated group showed decreased numbers of iNOS-expressing macrophages (Figure 4A, B) with increased populations of arginase 1-expressing macrophages (Figure 4D, E), compared with the rat IgG control group. These differences were significant from 1 to 7 days post-injury (Figure 4C, F). Similarly, the MR16-1-treated group showed decreased numbers of CD16/32-expressing macrophages (Figure 4G, H) and increased numbers of CD206-expressing macrophages (Figure 4J, K) compared with the rat IgG control group. These differences were significant from 3 to 14 days post-injury (Figure 4I, L). No significant differences were detected in macrophage polarization between the MR16-1-treated group and rat IgG control group at 14 days after injury.

### MR16-1 treatment inhibited interferon (IFN)-γ production by neutrophils and increased interleukin (IL)-4 expression in microglia and macrophages

**Figure 5:**
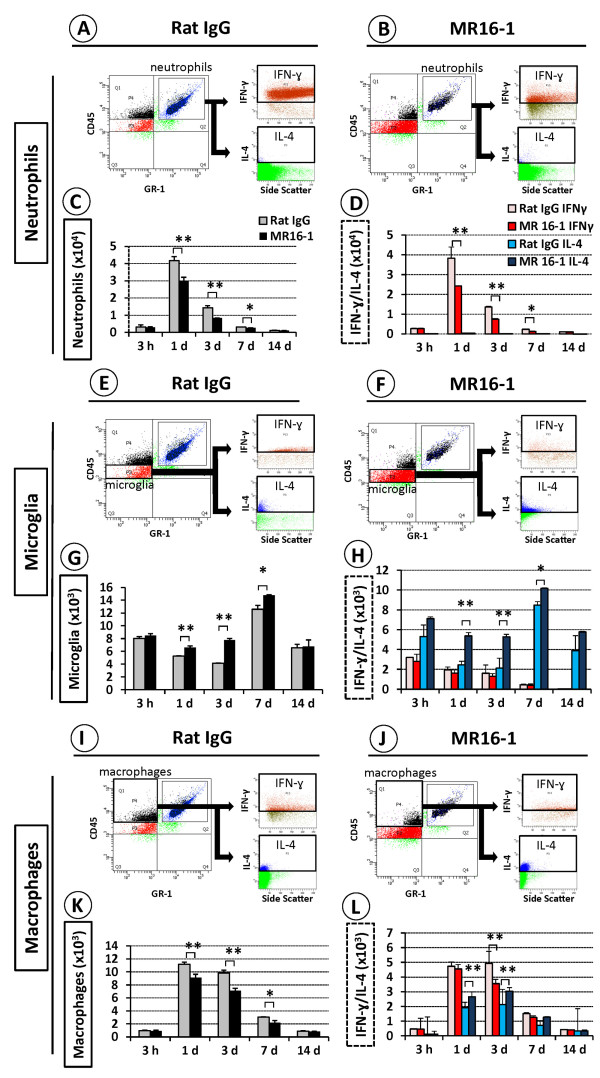
**Semi-quantitative flow-cytometry analysis of interferon (IFN)-γ and interleukin (IL)-4 levels in neutrophils, microglia and macrophages after MR16-1 treatment**. Representative flow-cytometry plots at 1 day post-injury showed large numbers of neutrophils positive for intracellular IFN-γ in (**A**) the rat IgG control group with (**B**) smaller numbers in the MR16-1-treated group. (**C**, **D**) The numbers of neutrophils and their levels of IFN-γ, but not IL-4, were significantly lower in the MR16-1-treated group compared with the control group from 1 to 7 days post-injury. (**E**, **F**) Representative data at 3 days post-injury showed that the MR16-1-treated group had larger numbers of microglia and greater positivity for IL-4 compared with the rat IgG control group, and this difference was significant from 1 day to 7 days post-injury. (**G**, **H**) However, there was no significant difference in IFN-γ expression. (**I, J, K**) At 3 days post-injury, the total number of macrophages was lower in (**I, J**) the MR16-1-treated group compared with (**K**) the control group, and this difference was significant from 1 to 7 days post-injury, with increased IL-4 expression at days 1 and 3, and (**L**) decreased expression of IFN-γ. (**C**, **D**, **G**, **H**, **K **and **L**) Data are expressed as mean ± SD; n = 5 for each group. (**C**, **G**, **K**) paired *t*-test; (**D**, **H**, **L**) ANOVA test; *P <0.05, **P <0.01.

Flow-cytometry analysis showed infiltration of large numbers of IFN-γ-positive neutrophils in the injured SC at 1 day after injury in the rat IgG control group (Figure 5A), but a significantly lower number in the MR16-1-treated group (Figure 5B). The decrease in neutrophil numbers in the MR16-1-treated group versus the rat IgG control group was significant from 1 to 7 days post-injury (Figure 5C). Furthermore, the number of IFN-γ-expressing neutrophils was also significantly different between the two groups throughout the same period (Figure 5D).

Significantly larger numbers of microglia were present in the MR16-1-treated group at 3 days after injury (Figure 5F) than in the rat IgG control group (Figure 5E). The numbers of microglia that stained positively for intracellular IL-4 were also increased in the MR16-1-treated group compared with the rat IgG control group, and this was significant from 1 to 7 days post-injury (Figure 5G, H). The number of macrophages infiltrating the SC after injury was reduced in the MR16-1-treated group compared with the rat IgG control group (Figure 5I, J), which was significant from 1 to 7 days post-injury (Figure 5K). Furthermore, within the macrophage population, the level of intracellular IL-4 detected at days 1 and 3 after injury was higher, in the MR16-1-treated group compared with the rat IgG control group, whereas intracellular IFN-γ at 3 days after injury was lower (Figure 5L). However, there were no significant differences in the numbers of the different cell populations studied or in their cytokine protein levels after 14 days post-injury.

### MR16-1 treatment changed the predominant phenotype of macrophages in the injured spinal cord, and promoted the phagocytic and digestive activities of macrophages

**Figure 6:**
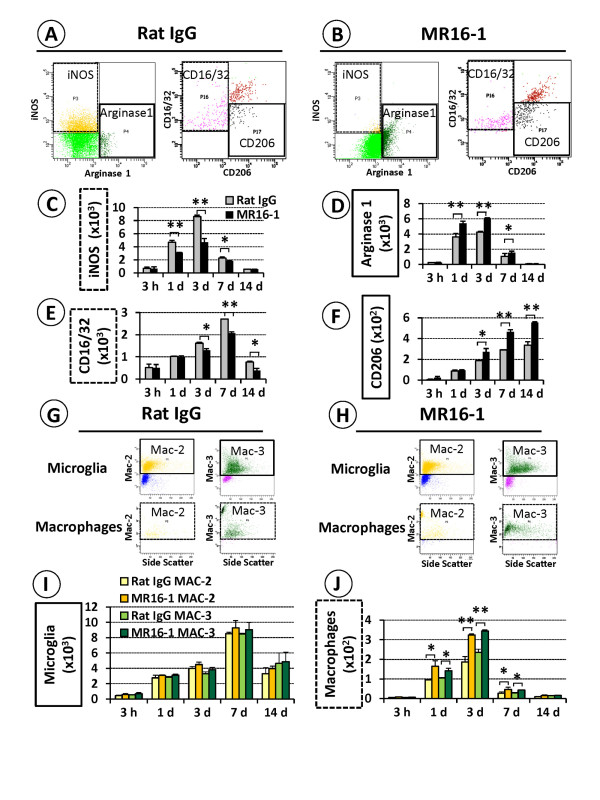
**Semi-quantitative flow-cytometry analysis of phagocytic and digestive activities of alternatively activated macrophages after MR16-1 treatment**. (**A**, **B**) Representative flow-cytometry data at 3 days post-injury identified larger numbers of iNOS-positive and CD16/32-positive macrophages in the injured SC of the rat IgG control group, compared with a lower number of such macrophages with an increased arginase 1-positive and CD206-positive sub-population in the MR16-1-treated group. (**C, D**) The differences in the relative preponderance of iNOS-positive and arginase 1-positive macrophages between the control and treatment group were significant from 1 to 7 days post-injury, whereas (**E**, **F**) the differences in CD16/32-positive and CD206-positive macrophages were significant from 3 days post-injury. (**G, H**) Microglia of both groups showed no major change in Mac-2 and Mac-3 expression; however, the number of Mac-2 and Mac-3-positive cells within the population of arginase 1-positive macrophages was significantly larger in the MR16-1-treated group than in the rat IgG control group. (**I**) There was no major difference in Mac-2 and Mac-3 expression in microglia, whereas (**J**) arginase 1-positive macrophages of the MR16-1-treated group showed enhanced expression of both antigens from 3 to 7 days post-injury. (**C-F**, **I**, **J**) Data are expressed as mean ± SD; n = 5 for each group;(**C**, **D**, **E, F**) paired *t*-test; (**I**, **J**) ANOVA. *P <0.05, **P <0.01

Flow-cytometry analysis showed that MR16-1 treatment was associated with a marked shift from an iNOS-positive and CD16/32-positive to an arginase 1-positive and CD206-positive macrophage population after injury (Figure 6A, B), matching the results of immunostaining. There was a significant decrease in the number of iNOS-positive macrophages and a substantial increase in the number of arginase 1-positive macrophages in the MR16-1-treated group compared with the rat IgG control group, and these differences were significant from 1 to 7 days post-injury (Figure 6C, D). A similar shift in the predominant phenotype of macrophages, from CD16/32-positive to CD206-positive, was also seen in the MR16-1-treated group versus rat IgG controls from 3 up to 14 days post-injury (Figure 6E, F).

Whereas microglia showed strong expression of Mac-2 and Mac-3, there was no significant difference between the microglia populations of the MR16-1-treated and rat IgG control groups (Figure 6G, I). However, a more detailed analysis of the cell populations showed that the arginase 1-positive macrophages of the MR16-1-treated group had enhanced positivity for Mac-2 and Mac-3 compared with the rat IgG control group, and these differences were significant from 1 up to 7 days post-injury (Figures 6H, J). No further significant differences were detected in macrophage polarization or antigen expression between the MR16-1-treated group and rat IgG control group after 14 days post-injury.

## Discussion

IL-6 is a multifunction cytokine crucial for T/B-cell differentiation and proliferation, immunoglobulin secretion, acute phase protein production, and macrophage/monocyte function [58]. In the pathophysiology of SCI, IL-6 is considered to be a pro-inflammatory cytokine that triggers secondary injury [33-35]. Once IL-6 is released, it binds to the membrane-bound IL-6R in an IL-6/IL-6R complex that associates with gp130 to exert a signal into cells [43]. MR16-1 is a rat anti-mouse IL-6R antibody that competitively inhibits the binding of IL-6 to IL-6R dose-dependently, has a half-life of about 3 days in mice, and exhibits anti-inflammatory properties in rheumatoid arthritis [59] and SCI [43,44]. In the present study of SCI, the MR16-1-treated group had smaller injury sites with less connective-tissue formation; these findings correlate with the blockade of reactive astrogliosis reported previously [44]. Increased myelin sparring and an enhanced SC repair process, as shown by an increased prevalence of NF-H-positive and GAP-43-positive fibers at 42 days post-injury, were also seen, and were probably the consequence of increased neuronal survival in response to a diminished inflammatory cascade, as a result of IL-6/IL-6R disengagement [52]. In addition, MR16-1 treatment improved locomotor BMS score from 7 days after SCI compared with the control groups, suggesting anatomical improvement, as reported by previous researchers [44,45,52]. We also found that MR16-1 treatment reduced the levels of the Th1 cytokines IFN-γ and TNF-α, with a parallel increase in levels of the Th2 cytokines IL-4 and IL-13 at the site of the spinal lesion in the acute phase after SCI. Hence, we hypothesized that a temporal blockade of IL-6 signaling by MR16-1 treatment changed the profile of cytokines present in the injured SC into an alternative macrophage-activating environment. In agreement with previous research, significant increases in IL-4 and IL-13 levels [21,24,28,29] and simultaneous reduction in IFN-γ and TNF-α levels would promote the formation of alternatively activated macrophages and inhibit that of the classically activated macrophages [19].

IFN-γ is mainly produced by blood-derived cells (neutrophils, macrophages, natural killer (NK) cells and T cells), while microglia in the CNS have also been reported to express IFN-γ [40,60]. The access of those blood cells to the SC after injury may be mediated by IL-6 through the IL-6R [37]. Therefore, MR16-1 treatment may restrict the initial access of blood cells to the injured spinal cord, hence leading to a reduction in IFN-γ level [61]. IFN-γ has been reported to have harmful effects in the CNS, including reduced proliferation of neuronal progenitor cells and increased apoptosis [41]. Conversely, other groups have described beneficial effects for IFN-γ administration in the CNS mediated through reduced chondroitin sulfate proteoglycan (CSPG) expression in reactive astrocytes, and increased expression of GDNF and IGF-1 [62,63]. Although it is clear that further exploration is needed into these different effects of IFN-γ, it is possible that IL-6 blockade may have elicited beneficial effects by decreasing IFN-γ in both conditions, as MR16-1 treatment blocks reactive astrogliosis and their CSPG expression [44], and also increases the production of neurotrophic factors by alternately activated macrophages [24,30]. Because this blockade in IL-6 signaling is temporal, a second wave of blood cells (probably NK cells and T cells) would be able to access the site of injury, which may in turn lead to a corresponding increased expression of IFN-γ, a theory that fits our immunoblot results. TNF-α mRNA, mainly from astrocytes, can be detected early after SCI, with a first peak after 1 hour [3]. IL-6 regulates the expression and secretion of TNF-α [64], which may explain the lower levels of TNF-α seen in the MR16-1-treated group compared with the control groups, possibly through inhibition of TNF-α expression in astrocytes [44].

Although macrophages and neutrophils are also thought to express IL-4 [48,49], microglia are considered the most important source of IL-4 and IL-13 in the acute phase after SCI [46,47], with increased expression of these cytokines at the peak of microglia activation [7], and subsequent reduction in cytokine levels associated with the death of these cells [6]. Our immunoblot analysis identified increased levels of both IL-4 and IL-13 after MR16-1 treatment compared with control levels, which probably correlated to the increased survival of microglia associated with the attenuation of the inflammatory cascade [53].

Hematogenous macrophages and microglia are major players in the inflammatory pathology of SCI [7,65]. Microglia are activated immediately after injury, leading to cell recruitment to the injury site. Microglia are believed to be relatively beneficial for SC repair, because of their high phagocytic activity and expression of various neurotrophic factors [45]. However, they are also reported to be incapable of replacing the roles of macrophages [66], and our results showed no significant difference between the microglia populations in the MR16-1-treated group and the rat IgG control group, in agreement with the previously reported data [45]. In fact, there are contradictory reports of functional recovery after SCI that correlated to both depletion and augmentation of macrophage populations, making such a therapeutic approach controversial [11,14,15]. Subsequent studies have correlated such divergent effects to the presence of different macrophages populations with contrasting functions [24]; classically activated macrophages (M1 phenotype) are the predominant type after SCI and have deleterious effects on the injured tissues, whereas alternatively activated macrophages (M2 phenotype) have only a short-term response, disappearing within 3 to 7 days after injury [21]. That sequence of events may be partially responsible for the lack of functional recovery after SCI [25].

Identification of macrophages using immunofluorescence labeling of specific markers allowed a more accurate phenotypic characterization of the different types of cells present, with the only limitation of this method being that it was a semi-quantitative analysis. Other researchers have reported that classically activated macrophages, producing pro-inflammatory cytokines and oxidative metabolites, are predominant at the site of injury and surrounding tissue after SCI [21]. We found a similar distribution and cytokine profile of classically activated macrophages after SCI. However, our analysis using double immunostaining showed that MR16-1 treatment reduced the population of iNOS-positive, CD16/32-positive cells (M1 phenotype) and promoted the population of arginase 1-positive, CD206-positive cells (M2 phenotype) at the site of the lesion in the acute phase after SCI. Other studies have reported that iNOS is the first M1 phenotypic marker upregulated in the classic activation pathway in the acute phase after injury, whereas arginase 1 is an early indicator for the alternative activation pathway and M2 macrophages [21]. CD16/32 and CD206 expression are also phenotypic hallmarks of M1 and M2 macrophages, respectively, seen mainly in the sub-acute phase of SCI [6]. CD206 correlates with active endocytosis and a fully activated phagocytic phenotype [24]. Our findings are in agreement with the reported sequences of the presence of M1 and M2 phenotypic markers after SCI, in which iNOS and arginase 1 were reported to reach peak values around 3 days and CD16/32 and CD206 peaked at 7 days post-injury. One point of interest for future studies will be to determine the source of these cell populations, which could be either resting macrophages after activation (that is, resident microglia in the CNS) or hematogenous macrophages. The use of our model is limited because both cells become morphologically indistinguishable by 1 week after activation, and there is no available cell marker that can specify their initial origin [6,31].

The results of flow cytometry showed relatively larger numbers of IFN-γ-overexpressing neutrophils and macrophages in the rat IgG control group compared with the MR16-1-treated group, similar to the results of previous studies [32,38]. These results are in agreement with previous studies that reported the important role of IL-6 in the induction of chemokines and leukocyte recruitment after SCI [18,61]. MR16-1 treatment was reported to reduce the expression of such factors [45], a finding that might be responsible for the diminished CD11b^positive ^population seen after MR16-1 treatment in our study, as reported previously [44]. IFN-γ expression is also upregulated by TNF-α [60], which in our study was decreased after MR16-1 treatment. Previous studies identified IL-4 as one of the most important factors to generate alternatively activated macrophages *in vitro *and *in vivo *in neural tissues [67], and identified microglia as the main source of IL-4 in the CNS [46]. We found that MR16-1 treatment significantly increased the microglial population, with a corresponding increase in IL-4 levels by up to twofold. These results are in agreement with studies that reported an increase in the levels of colony-stimulating factors after MR16-1 treatment [45], which are responsible for the proliferation of microglia in rodents *in vivo *[68]. We also saw significantly higher numbers of alternatively activated macrophages in the MR16-1-treated group compared with controls up to 14 days post-injury, with increased functional recovery. Alternatively activated macrophages have potentially beneficial effects by increasing nerve growth *in vivo*, as seen by he enhanced growth of adult dorsal root ganglion axons and the promotion of cAMP in the growing axons through enhanced expression of brain-derived neurotrophic factor [69].

In addition, in our study we found enhanced phagocytic activity for these macrophages, as identified by positivity for Mac-2 and Mac-3. A previous study reported no change in the macrophage expression of these proteins after MR16-1 treatment [45]; however, the macrophages were analyzed as a single population in that study, whereas our study was performed on sub-populations of macrophages. Mac-2 correlates with increased phagocytic activity and debridement of scar tissue, whereas Mac-3 typically correlates with the presence of digestive enzyme-producing lysosomes and endosomes [70,71]. The increased phagocytic abilities of these macrophages provide another possible mechanism for the reduced scar tissue formation found in the MR16-1-treated group, together with possible reduction in reactive astrogliosis, through reduced levels of inflammatory cytokines [44]. In fact, this is one limitation of this model, because we could not determine whether the regeneration was due to the direct action of the alternatively activated macrophages or to other effects of the blockade of IL-6.

Based on the results of this study, we considered that administration after SCI of the IL-6 blocking antibody MR16-1 promotes changes in the microenvironment of the injury site, reducing TNF-α expression and its effects, restraining the entry of neutrophils into the injured spinal cord, reducing the levels of IFN-γ (as a result of low production of recruiting cytokines), and augmenting IL-4-expressing and IL-13-expressing microglia [33,60]. The high levels of these Th2 cytokines (IL-4, IL-13) will activate hematogenous macrophages and resident macrophages through the IL-4Rα/JAK/STAT signaling pathway into alternatively activated M2 macrophages, whereas the low levels the of Th1 cytokines (TNF-α, IFN-γ) will prevent the macrophages shifting back into an M1 phenotype associated with classic activation [17,24]. Such alternatively activated macrophages have increased phagocytic abilities because of the presence of endosomes/lysosomes and pinocytic structures with digestive enzymes [21,22], efficiently removing scar tissue and growth inhibitors present in myelin debris, and allowing axonal regeneration with subsequent locomotor recovery [29,30].

The use of MR16-1 antibody *in vivo *seems promising, as it produced lasting effects that did not seem to be related to the half-life of the drug itself, and exerted its anti-inflammatory properties after a single dose. A humanized version of MR16-1 is already available for the treatment of rheumatoid arthritis and approved by the US FDA and the European Union ((as Actemra^® ^and RoActemra^®^, respectively; Hoffman-La Roche, Nutley, NJ, USA), thus MR16-1 treatment could be a feasible option for translational research of therapeutic approaches for SCI in humans in the near future. It is our opinion that quantitative evaluation of the production of neurotrophic factors by macrophages after IL-6 blockade and analysis of the effects of the blockade of other Th1 cytokines, such as TNF-α [54], would enhance our understanding of the potential effects of MR16-1 treatment.

## Conclusion

A temporal blockade of IL-6 signaling by MR16-1 antibody could promote the generation of alternatively activated macrophages (M2 phenotype), and thus modify the inflammatory response after SCI to promote SC regeneration with functional recovery.

## Abbreviations

APC: allophycocyanin; BMS: Basso Mouse Scale; cAMP: cyclic adenosine monophosphate; CNS: central nervous system; CSPG: chondroitin sulfate proteoglycan; DAPI: 4',6-diamidino-2-phenylindole; DMEM: Dulbecco's modified Eagle's Medium; FDA: United States Food and Drug Administration; GAP-43: growth-associated protein-43; IL: interleukin; INF-γ: interferon-gamma; iNOS: inducible nitric oxide synthase; JAK: Janus-activated kinase; NK: natural killer; LFB: Luxol fast blue; Mac: macrophage antigen; NF-H: neurofilament heavy 200 kDa; PBS: phosphate-buffered saline; SCI: spinal cord injury; STAT: signal transducer and activator of transcription; Th: T helper; TNF-α: tumor necrosis factor-alpha.

## Competing interests

The authors declare that they have no competing interests.

## Authors' contributions

ARG carried out most of the experiments and drafted the manuscript. KU was responsible for the experimental design and data analysis. HN and SW helped with immunohistochemistry and flow-cytometry analysis, and participated in the evaluation of BMS. MN and WEB J revised the paper and provided technical support. HB participated in the data analysis, conceptualization, and final edition of the paper. All authors read and approved the final manuscript.

### Disclaimer

No author has any financial ties to commercial parties.

## Supplementary Material

Additional file 1 **Immunoblot analysis of interleukin (IL)-6 and IL-6 receptor (IL-6R) after treatment with MR16-1**. (**A, B**) IL-6 was persistently upregulated in the control groups, with peak expression at 3 hours post-injury, and the difference from the MR16-1-treated group was significant up to 7 days post-injury. (**C, D**) Upregulation of IL-6R in the control groups with peak expression at 1 day, and significant difference from the treatment group up to 7 days post-injury. There was no difference in cytokine expression between the saline and rat IgG control groups. (**B**,**D**) Each graph represents the band intensity relative to that of β-actin. Data are expressed as mean ± SD, n = 3 for each group. *P <0.05, **P <0.01 by ANOVA. Click here for file

Additional file 2 **Controls used in flow-cytometry analysis**. (**A**-**E**) Mixed preparations of injured and control spinal cord samples were used as negative controls; these samples were used without added fluorescent-conjugated antibodies to set the gates through all the scatter plots to be used in the flow-cytometry experiment, adjusting the high voltages (HVs) per detector, making sure that the negative population was clearly off the axis in every channel. (**F**-**P**) Mixed preparations of the same samples with a single added fluorescent-conjugated antibody were used as positive controls to adjust the fluorescence compensation in each channel, eliminating signal overlapping. Proper compensation was considered achieved when, in every given channel, the positive controls had the same mean of the negative controls. (**Q**) Light-scatter plot (abscissa: forward scatter; ordinate: side scatter) of a mixed sample of injured and control spinal cords was used to define the region of interest to be studied (black triangle area). (**R**) Samples of representative naive spinal cord (SC) were used to collect baseline information about the populations of microglia (red squares: CD11b^high^, CD45^low^, GR-1^negative^), macrophages (black squares: CD11b^high^, CD45^high^, GR-1^negative^) and neutrophils (blue squares: CD11b^high^, CD45^high^, GR-1^high^). Few neutrophils and macrophages were detected in the naive SC samples. (**S**) Samples from a representative sham-injured sample at 3 days showed a slight increase in the number of cells in the region of interest, with a subsequent increase in all the populations studied. (**T**) Density plots of representative SC samples at 3 days post-injury from the rat IgG control group showed a robust increase in the number of cells present in the region of interest, representing massive invasion of macrophages and neutrophils, together with an increased microglial population after injury. (**U**) SC samples from MR16-1-treated group at 3 days post-injury showed a similar increase in the region of interest, and further analysis identified an enhanced number of microglia together with fewer macrophages and neutrophils, relative to the rat IgG control group. Click here for file

Additional file 3 **Cell identification by flow cytometry**. **(A) **CD11b ^positive ^cells from the spinal cord (SC) samples were analyzed and fractioned in three different populations: CD11b^negative^, CD11b^low ^and CD11b^high^. The CD11b^high ^population was sub-fractioned based on the expression of CD45 and GR-1 into three major sub-populations; CD45^high^/GR-1^high ^neutrophils, CD45^high^/GR-1^negative ^macrophages and CD45^low^/GR-1^negative ^microglia. The expression of interferon (IFN)-γ and interleukin (IL)-4 was assessed in each population. (**B**) CD11b^high ^cells in the SC were sub-fractioned into CD45^high^/GR-1^negative ^(macrophages) and CD45^low^/GR-1^negative ^(microglia). The phenotype of such macrophages was confirmed by their expression of inducible nitric oxide synthase (iNOS) or CD16/32 (classically activated macrophages) and arginase 1 or CD206 (alternatively activated macrophages). (**C**) The expression levels of macrophage antigen (Mac)-2 and Mac-3 were quantified in macrophages positive for arginase 1, and in the microglia of both groups. Click here for file
