# Supplementary figures and images for: Blockade of interleukin-6 signaling inhibits the classic pathway and promotes an alternative pathway of macrophage activation after spinal cord injury in mice
Source: J Neuroinflammation. 2012 Feb 27;9:40. doi: 10.1186/1742-2094-9-40 (PMC3310810; doi:10.1186/1742-2094-9-40)

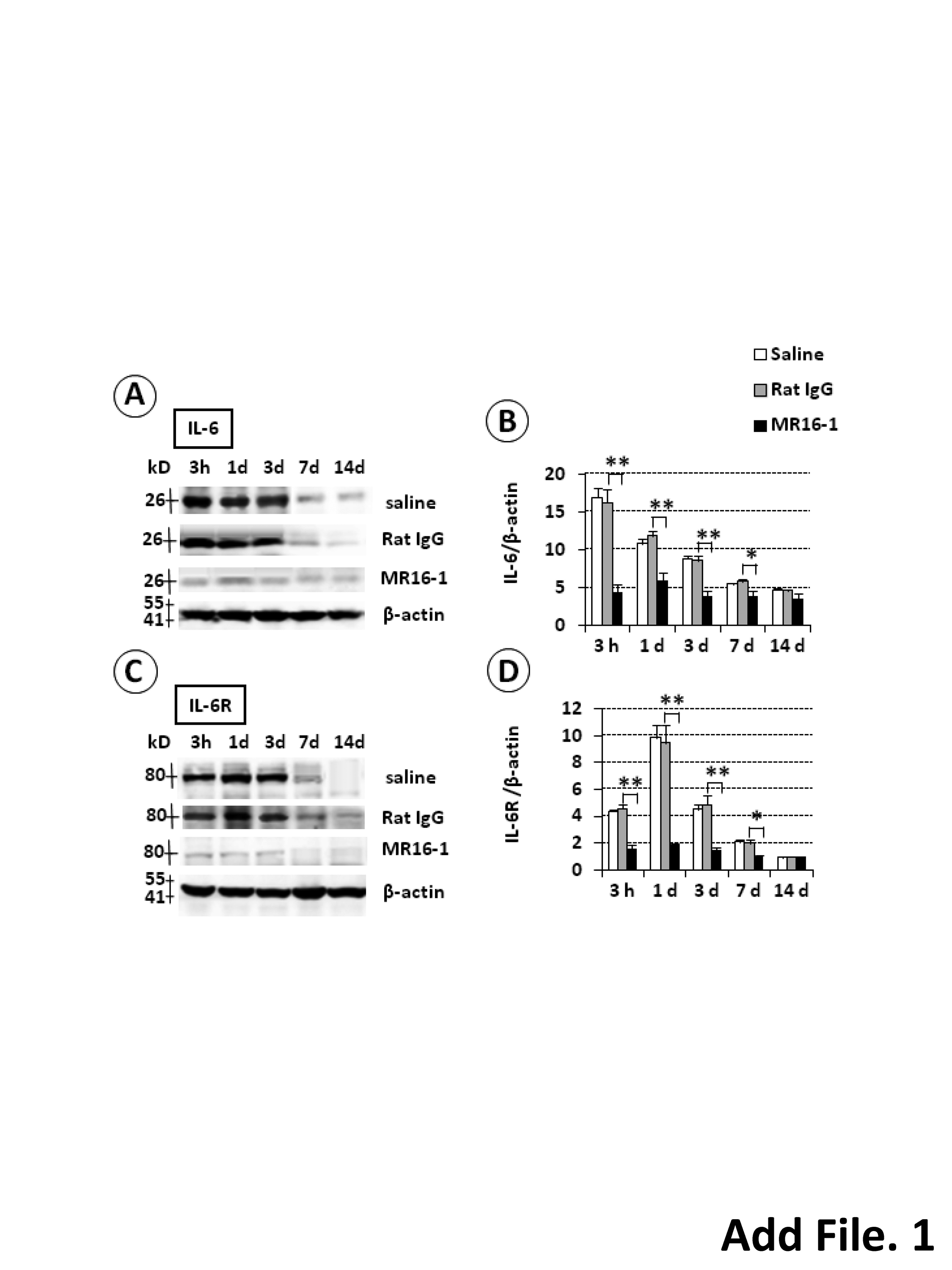

Supplement: Additional file 1 — Immunoblot analysis of interleukin (IL)-6 and IL-6 receptor (IL-6R) after treatment with MR16-1. (A, B) IL-6 was persistently upregulated in the control groups, with peak expression at 3 hours post-injury, and the difference from the MR16-1-treated group was significant up to 7 days post-injury. (C, D) Upregulation of IL-6R in the control groups with peak expression at 1 day, and significant difference from the treatment group up to 7 days post-injury. There was no difference in cytokine expression between the saline and rat IgG control groups. (B,D) Each graph represents the band intensity relative to that of β-actin. Data are expressed as mean ± SD, n = 3 for each group. *P <0.05, **P <0.01 by ANOVA. [file 1742-2094-9-40-S1.TIFF]

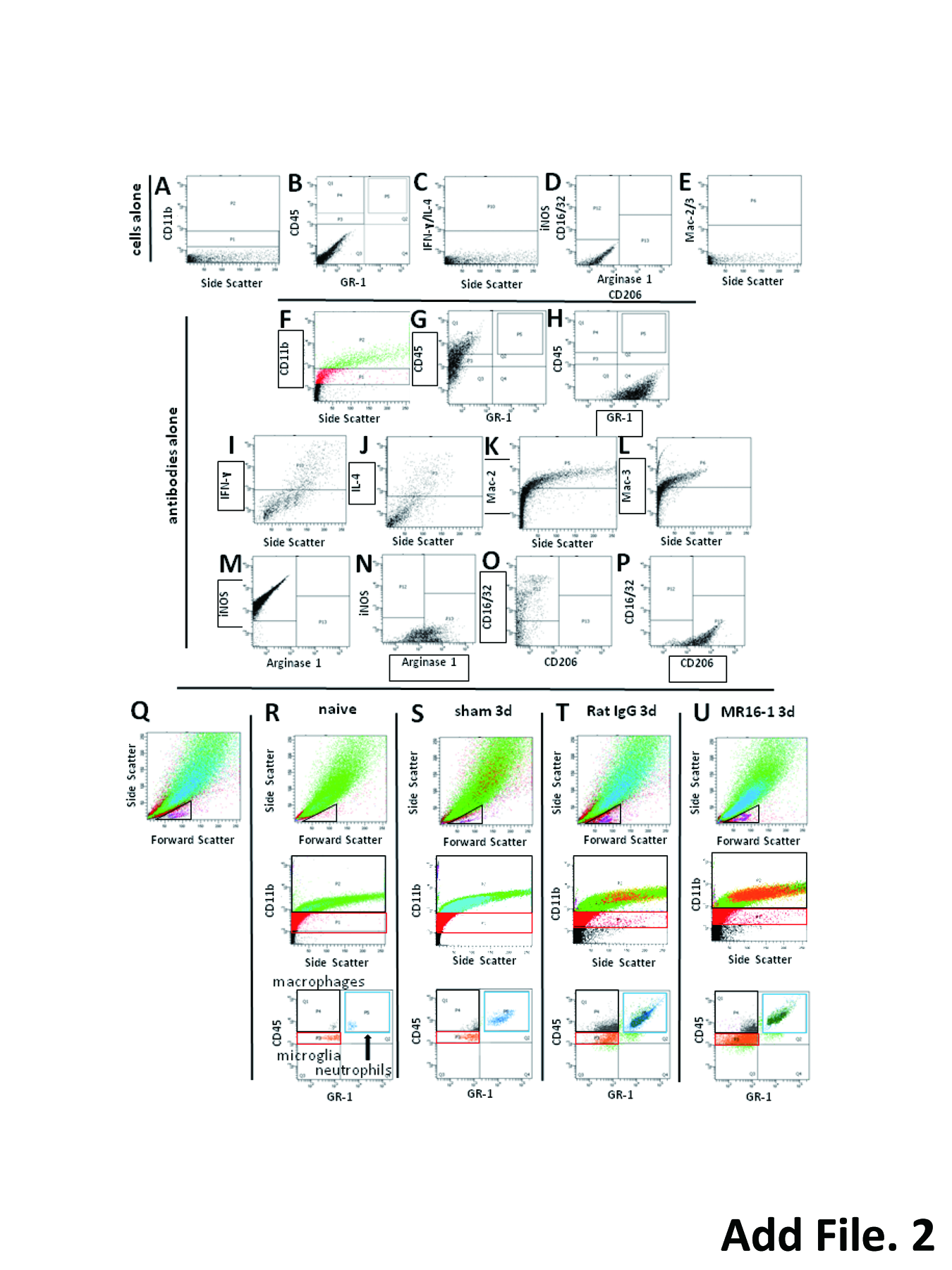

Supplement: Additional file 2 — Controls used in flow-cytometry analysis. (A-E) Mixed preparations of injured and control spinal cord samples were used as negative controls; these samples were used without added fluorescent-conjugated antibodies to set the gates through all the scatter plots to be used in the flow-cytometry experiment, adjusting the high voltages (HVs) per detector, making sure that the negative population was clearly off the axis in every channel. (F-P) Mixed preparations of the same samples with a single added fluorescent-conjugated antibody were used as positive controls to adjust the fluorescence compensation in each channel, eliminating signal overlapping. Proper compensation was considered achieved when, in every given channel, the positive controls had the same mean of the negative controls. (Q) Light-scatter plot (abscissa: forward scatter; ordinate: side scatter) of a mixed sample of injured and control spinal cords was used to define the region of interest to be studied (black triangle area). (R) Samples of representative naive spinal cord (SC) were used to collect baseline information about the populations of microglia (red squares: CD11bhigh, CD45low, GR-1negative), macrophages (black squares: CD11bhigh, CD45high, GR-1negative) and neutrophils (blue squares: CD11bhigh, CD45high, GR-1high). Few neutrophils and macrophages were detected in the naive SC samples. (S) Samples from a representative sham-injured sample at 3 days showed a slight increase in the number of cells in the region of interest, with a subsequent increase in all the populations studied. (T) Density plots of representative SC samples at 3 days post-injury from the rat IgG control group showed a robust increase in the number of cells present in the region of interest, representing massive invasion of macrophages and neutrophils, together with an increased microglial population after injury. (U) SC samples from MR16-1-treated group at 3 days post-injury showed a similar increase in the region of interest, [file 1742-2094-9-40-S2.TIFF]

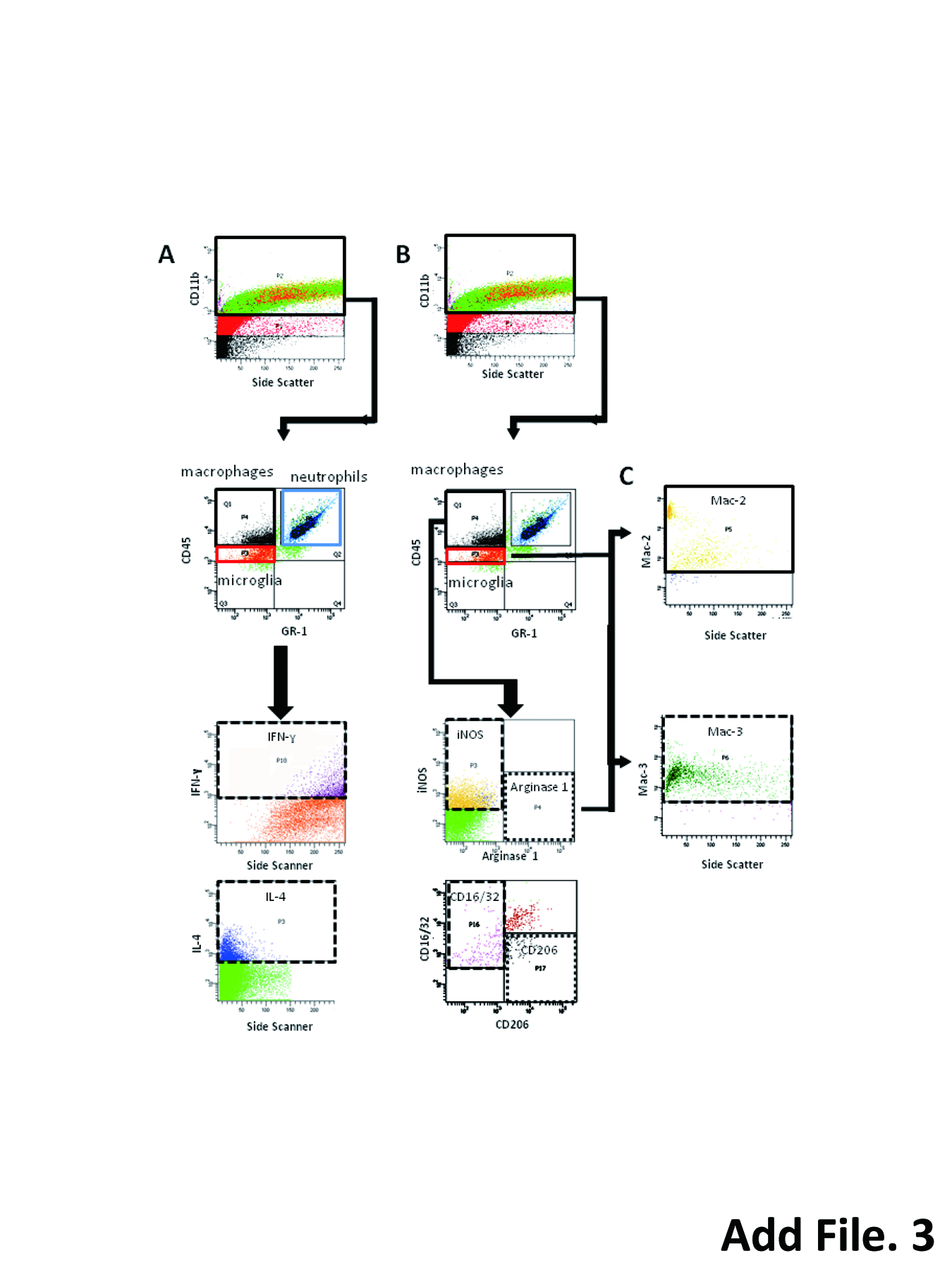

Supplement: Additional file 3 — Cell identification by flow cytometry. (A) CD11b positive cells from the spinal cord (SC) samples were analyzed and fractioned in three different populations: CD11bnegative, CD11blow and CD11bhigh. The CD11bhigh population was sub-fractioned based on the expression of CD45 and GR-1 into three major sub-populations; CD45high/GR-1high neutrophils, CD45high/GR-1negative macrophages and CD45low/GR-1negative microglia. The expression of interferon (IFN)-γ and interleukin (IL)-4 was assessed in each population. (B) CD11bhigh cells in the SC were sub-fractioned into CD45high/GR-1negative (macrophages) and CD45low/GR-1negative (microglia). The phenotype of such macrophages was confirmed by their expression of inducible nitric oxide synthase (iNOS) or CD16/32 (classically activated macrophages) and arginase 1 or CD206 (alternatively activated macrophages). (C) The expression levels of macrophage antigen (Mac)-2 and Mac-3 were quantified in macrophages positive for arginase 1, and in the microglia of both groups. [file 1742-2094-9-40-S3.TIFF]
